# Supplementary material for: An amphipathic helix in Brl1 is required for nuclear pore complex biogenesis in S. cerevisiae
Source: eLife. 2022 Aug 24;11:e78385. doi: 10.7554/eLife.78385 (PMC9402233; doi:10.7554/eLife.78385)

Figure 5E

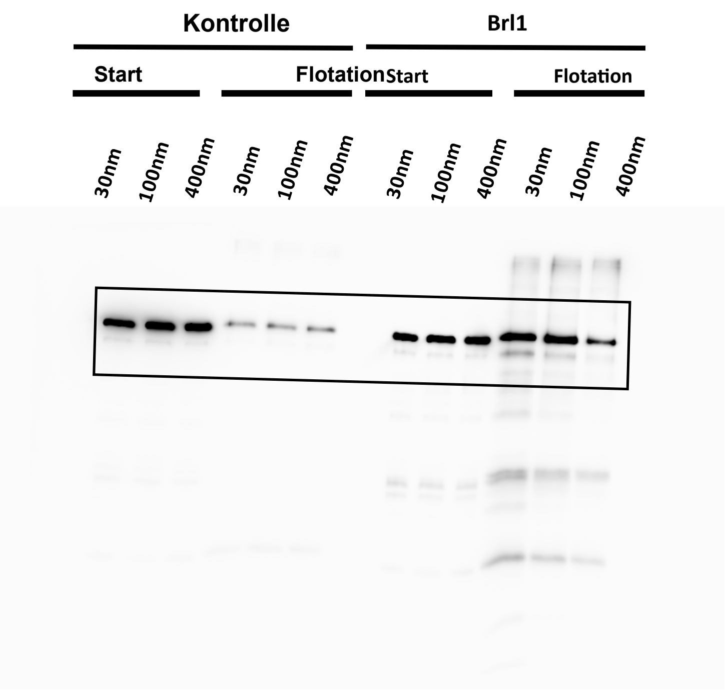

Figure 5F

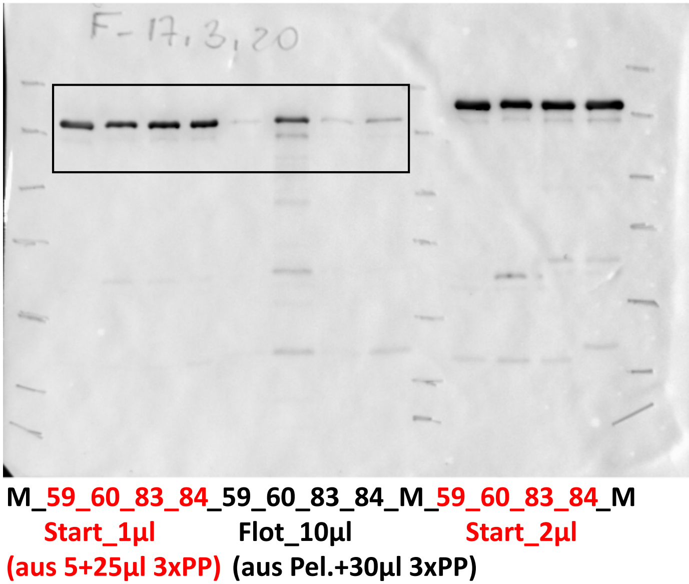

Figure 3—figure supplement 1B

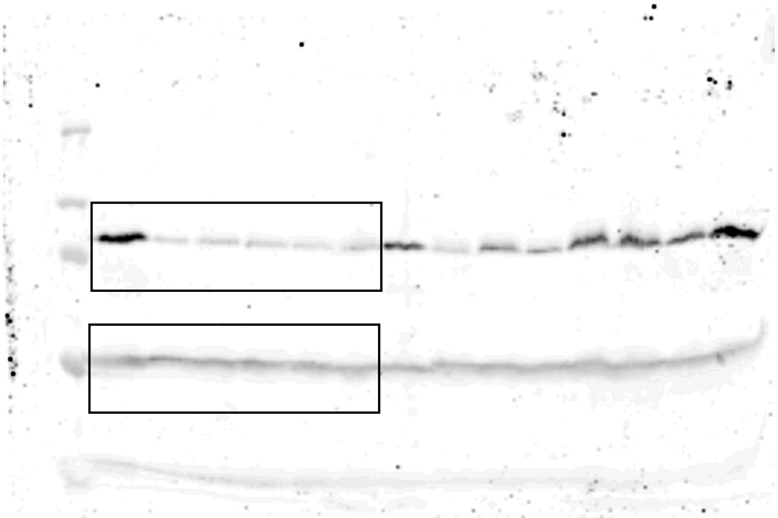

Supplement: Figure 3—figure supplement 1—source data 1. [file elife-78385-fig3-figsupp1-data1.pdf]
